# Assessing semantic interoperability in environmental sciences: variety of approaches and semantic artefacts

**DOI:** 10.1038/s41597-024-03669-3

**Published:** 2024-09-27

**Authors:** Cristina Di Muri, Martina Pulieri, Davide Raho, Alexandra N. Muresan, Andrea Tarallo, Jessica Titocci, Enrica Nestola, Alberto Basset, Sabrina Mazzoni, Ilaria Rosati

**Affiliations:** 1 National Research Council of Italy (CNR), Research Institute on Terrestrial Ecosystems (IRET), Lecce, 73100 Italy; 2 University of Salento, Department of Biological and Environmental Sciences and Technologies, Lecce, 73100 Italy; 3 LifeWatch Italy, Lecce, 73100 Italy; 4 LifeWatch ERIC, Lecce, 73100 Italy; 5 National Research Council of Italy (CNR), Institute for Sustainable Plant Protection (IPSP), Sesto Fiorentino, 50019 FI Italy

**Keywords:** Environmental sciences, Research management

## Abstract

The integration and reuse of digital research products can be only ensured through the adoption of machine-actionable (meta)data standards enriched with semantic artefacts. This study compiles 540 semantic artefacts in environmental sciences to: *i*. examine their coverage in scientific domains and topics; *ii*. assess key aspects of their FAIRness; and *iii*. evaluate management and governance concerns. The analyses showed that the majority of semantic artefacts concern the terrestrial biosphere domain, and that a small portion of the total failed to meet the FAIR principles. For example, 5.5% of semantic artefacts were not available in semantic catalogues, 8% were not built with standard model languages and formats, 24.6% were published without usage licences and 22.4% without version information or with divergent versions across catalogues in which they were available. This investigation discusses common semantic practices, outlines existing gaps and suggests potential solutions to address semantic interoperability challenges in some of the resources originally designed to guarantee it.

## Introduction

In the age of technology-driven and machine-assisted research, data are no longer only those gathered by humans through observations, laboratory/field experiments and hypothesis testing but also those arising in digital form (*e.g*. lines of code, software, or AI-generated data). In addition, with the ever-increasing automation and instrumentation advances, machines have become generators of data as well as analytical assistants, such as in the case of autonomous monitoring systems, remote sensing devices, sensors and so forth. All such types of digital data require to be properly interpreted and reused by different researchers and computers to be fully exploited^1^.

Semantic interoperability allows the exact transmission of the format and the meaning of research products as well as the precise exchange of information between parties^2^, hence, it represents one of the fundamental pillars of the FAIR principles^3^, a set of guidelines to improve the Findability, Accessibility, Interoperability, and Reusability of scientific data and other research products including workflows, software and semantic artefacts^4–6^.

The adoption of semantic artefacts is required to achieve semantic interoperability, to describe the meaning of data and relations among them. Semantic artefacts are machine-readable, -interpretable and -actionable formalisations of concepts that can be used and exchanged to encode and predictably decode information, thus enabling the discovery, integration and reuse of information by both humans and machines^7^. Semantic artefacts provide a framework for conflict resolution between terms developed independently across different disciplines or groups of interest.

Thus far, different terms may have been used to describe the same concept or, alternatively, the same term may has been used to express several concepts across different disciplines/communities, thereby impeding interoperability. For such a reason, despite semantic artefacts are created and used to align research products with the FAIR principles, their variety and heterogeneity are considered a source of lack of semantic interoperability^2^.

Semantic artefacts may have a broad range of formalisations, from loose sets of terms such as lists, glossaries, and categorisation schemes to higher-order logic constructs such as thesauri and ontologies^8,9^. As a consequence, they are built using different standard models (*e.g*. RDFS, OWL, SKOS) and serialisation schemes (*e.g*. XML, XML Schema, JSON, RDF/XML, OWL/XML, JSON-LD, Turtle, N-Triples, etc.)^7^.

Semantic artefacts are often stored and shared by means of semantic catalogues that facilitate their discoverability and access, support their management and use, and in some cases provide additional editing tools and services^10^. These catalogues can be registries, namely, metadata catalogues of semantic artefacts, and repositories that store and offer access to semantic artefacts metadata as well as to their content^2,7,8^. Even though these catalogues facilitate the discovery of and access to semantic artefacts in accordance to the FAIR principles, the distribution of such resources over multiple catalogues could, in some cases, lead to duplications and inconsistencies that compromise their long term sustainability.

Accounting for the value of semantic interoperability in interdisciplinary research, such as that of environmental sciences, an extensive collection and analysis of existing semantic artefacts in such a context can be critical to outline the state of the art of semantic interoperability and to highlight existing gaps.

This study was initiated in the framework of the project “Italian Integrated Environmental Research Infrastructures System” (ITINERIS), a multidisciplinary project coordinating a network of national nodes from 22 environmental Research Infrastructures (RIs). One of the main aims of the project is to build the Italian Hub of RIs providing centralised access to existing and novel FAIR data and related services (*e.g*. codes, software and any other digital research outputs; Digital Objects; DOs)^11^ used to study environmental processes in the atmosphere, marine, terrestrial biosphere, and geosphere land surface domains. The achievement of such an objective requires the utilisation of FAIR semantic artefacts that can facilitate the search and retrieval of interdisciplinary DOs available through the Hub and enable their reuse. This endeavour can accelerate the synthesis of existing scientific evidence and the generation of novel knowledge supporting decision-making to tackle current and expected environmental challenges^12^.

With this in mind, an extensive search of existing semantic artefacts was carried out starting from the semantic repositories used by the ITINERIS RIs. The search was then widened to include further semantic artefacts and catalogues focusing on the environmental domain.

The collection of semantic artefacts and their metadata was performed to *i*. examine their coverage in topics and environmental domains; *ii*. assess key properties that influence their findability, accessibility, interoperability and reusability; *iii*. evaluate the management, governance and long term sustainability of semantic artefacts available across multiple catalogues. Specifically, the analysis described in *ii*. consisted in the evaluation of 13 metadata properties associated with seven FAIR sub-principles considered relevant for publishing FAIR semantic artefacts^13^. The properties and associated FAIR sub-principles (in brackets) included in the analysis are: identifiers (F1), inclusion in semantic catalogues (F4), status (A1), formality level, language and format (I1), description (R1), usage licence (R1.1) and version (R1.2).

This study can provide useful insights to the ITINERIS RIs that still do not fully embrace semantic interoperability practices and support their future implementation. In addition, and beyond ITINERIS, this analysis is pivotal to emphasise the strengths and weaknesses of semantic artefacts in the field of environmental sciences and also to showcase existing gaps and challenges still limiting cross-domain semantic interoperability.

## Results

### Coverage: environmental domains and topics

**Fig. 1:**
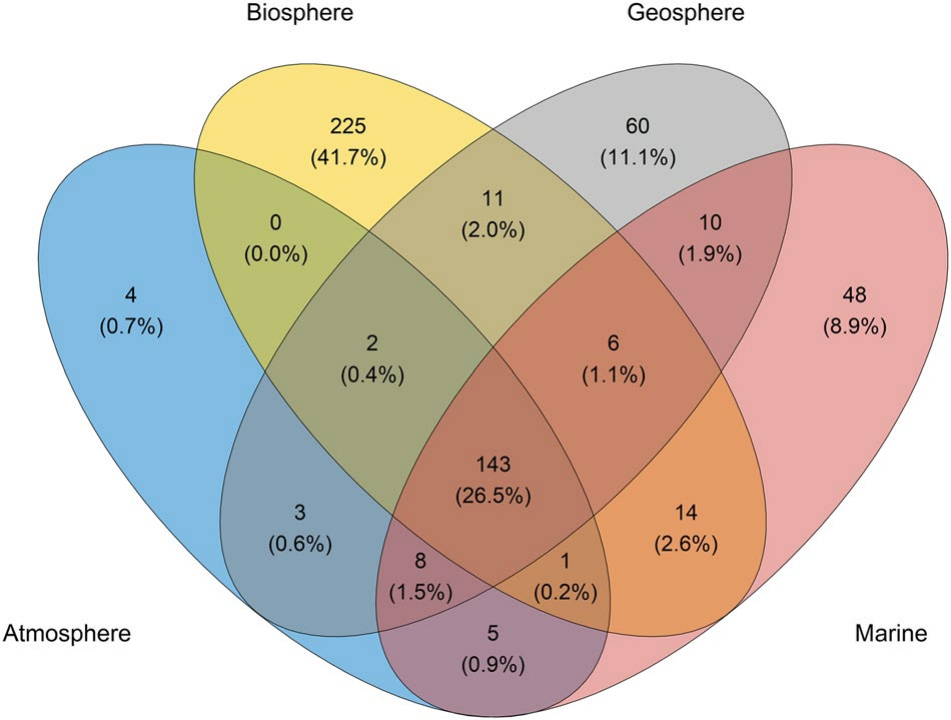
Venn diagram displaying the number and percentage (in bracket) of shared and unique semantic artefacts of the four environmental domains considered in this study according to the ITINERIS classification of environmental domains.

A total number of 540 semantic artefacts were compiled^14^ and classified, according to the ITINERIS project, in the four environmental domains. Specifically, 225 out of 540 resources provided terms exclusively for the terrestrial biosphere domain (41.7%; Fig. 1), 60 for the geosphere land surface domain (11.1%; Fig. 1), 48 for the marine domain (8.9%; Fig. 1), and four for the atmosphere domain (0.6%; Fig. 1). In addition, 143 semantic artefacts covered all environmental domains (26.5%; Fig. 1) and 60 concerned multiple domains (Fig. 1).

**Fig. 2:**
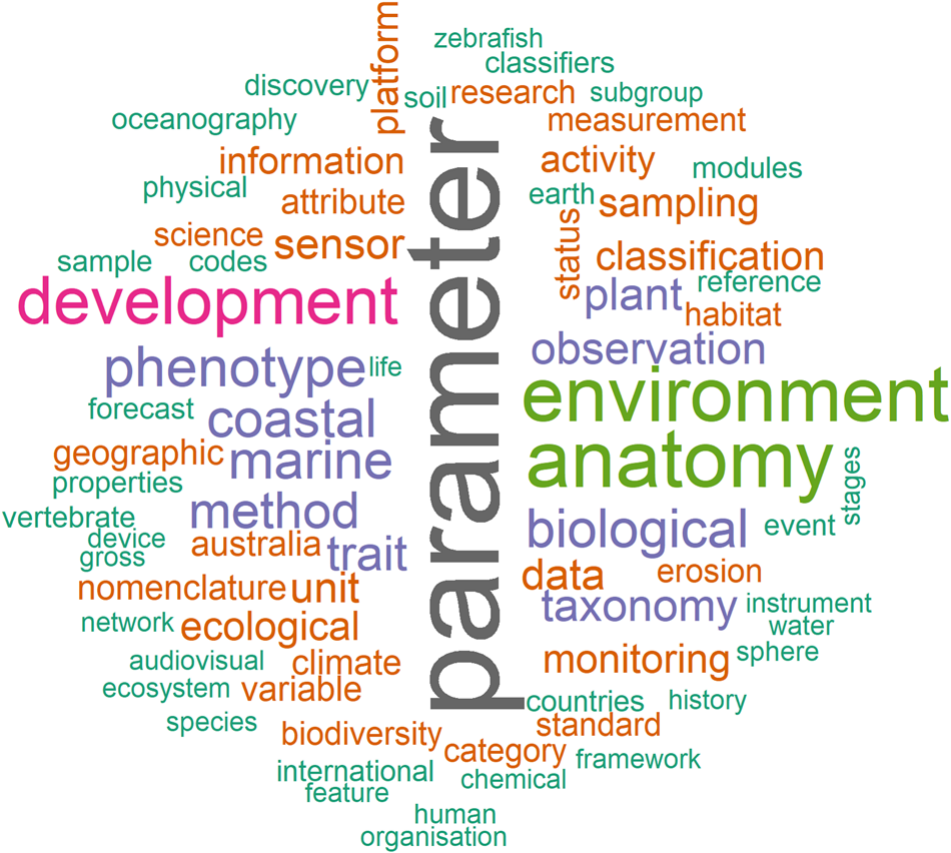
Word Cloud showing the most common terms used to describe the semantic artefacts included in this study. The image is the result of a text mining analysis performed on the titles of the compiled semantic artefacts.

A text mining analysis on semantic artefacts’ titles was carried out to identify the topics covered (Fig. 2). The results showed that the most frequent terms were parameter (*freq* = 43), anatomy (*freq* = 24), environment (*freq* = 23), development (*freq* = 19), phenotype (*freq* = 15), biological (*freq* = 14), coastal (*freq* = 14) and marine (*freq* = 14), method (*freq* = 13), trait (*freq* = 12), observation (*freq* = 11), plant (*freq* = 11), taxonomy (*freq* = 11), data (*freq* = 10), sensor (*freq* = 10), and unit (*freq* = 10).

### FAIRness of semantic artefacts

#### Findability: identifiers and semantic catalogues

The findability of semantic artefacts was evaluated by performing an analysis of identifiers (F1; mod:URI; dct:identifier) and of semantic catalogues in which the semantic artefacts were available (F4; schema:includedInDataCatalog).

All semantic artefacts were entities of the digital realm that can be identified with Uniform Resource Identifiers (URIs), Persistent Uniform Resource Locators (PURLs) or through Uniform Resource Locators (URLs) of the web pages in which they were available. In addition, 100 semantic artefacts were also minted with Digital Object Identifiers (DOIs).

**Fig. 3:**
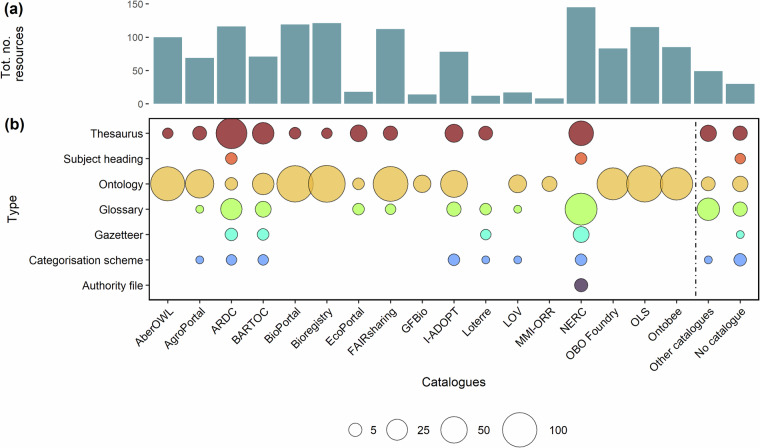
Distribution, number and type of semantic artefacts. The barplot (**a**) shows the total number of semantic artefacts per catalogue. The bubble chart (**b**) describes the distribution and the type of semantic artefacts across the 17 semantic catalogues considered in this study. In “Other catalogues” and “No catalogue” are shown respectively the number and the type of semantic artefacts found in catalogues not considered within this study and those not available in any catalogue.

As about their online distribution, 510 semantic artefacts were included in the semantic catalogues considered in this study (Table 1) and 30 were not (Fig. 3). Specifically, 145 semantic artefacts were available in the NERC Vocabulary Server, 121 in Bioregistry, 119 in BioPortal, 116 in Research Vocabularies Australia (ARDC), 115 in EMBL-EBI Ontology Lookup Service (OLS), 112 in FAIRsharing, 100 in AberOwl, 85 in Ontobee, 83 in Open Biological and Biomedical Ontology (OBO) Foundry, 78 in the I-ADOPT Catalogue of Terminologies, 71 in Basic Register of Thesauri, Ontologies and Classifications (BARTOC), 69 in AgroPortal, 18 in EcoPortal, 17 in Linked Open Vocabularies (LOV), 14 in the German Federation for Biological data terminology service (GFBio), 12 in Linked Open TERminology REsources (LOTERRE), and 8 in the Marine Metadata Interoperability Ontology Registry and Repository (MMI-ORR) (Fig. 3). Lastly, 49 semantic artefacts were available in catalogues not considered in this study (*i.e*. the TERN Linked Data Services, the United Nation TERMinology database [UNTERM], the INSPIRE Registry, the GBIF Repository of Schemas, and the ESIP Community Ontology Repository [ESIP COR]).

**Table 1:** List of semantic catalogues, in alphabetical order, used to gather semantic artefacts. The table includes the catalogues’ full names and acronyms with URLs, the type of catalogue (repository or registry), and the total number of semantic artefacts available.

Catalogue name (acronym)	Type	Semantic artefacts
AberOWL	Repository	1,422
AgroPortal	Repository	150
Basic Register of Thesauri, Ontologies and Classifications (BARTOC)	Registry	3,429
BioPortal	Repository	1,044
Bioregistry	Registry	1,650
NERC Vocabulary Server (NVS)	Repository	292
EcoPortal	Repository	25
FAIRsharing	Registry	833
German Federation for Biological data (GFBio)	Repository	29
I-ADOPT Catalogue of Terminologies	Registry	84
Linked Open TERminology REsources (LOTERRE)	Repository	65
Linked Open Vocabularies (LOV)	Repository	812
Marine Metadata Interoperability Ontology Registry and Repository (MMI-ORR)	Repository	330
Ontobee	Repository	262
Open Biological and Biomedical Ontology (OBO) Foundry	Registry	185
EMBL-EBI Ontology Lookup Service (OLS)	Repository	242
Research Vocabularies Australia (ARDC)	Repository	443

#### Accessibility: status and maintenance

The status and maintenance of semantic artefacts were analysed to evaluate their accessibility (A2; mod:status; owl:deprecated).

For 349 semantic artefacts the status of the resource was not explicitly defined, whereas, in all other instances, the status was specified as being in “Production” (*N* = 137), “Alpha” (*N* = 24), “Retired” (*N* = 13), “Beta” (*N* = 10), “Inactive” (*N* = 5), and “Uncertain” (*N* = 2). The first four status terminologies are standard states of software development life cycle, whereas “Inactive” and “Uncertain” are custom terminologies. Moreover, 13 semantic artefacts were declared as no longer maintained (*i.e*., deprecated) and 81 as maintained. In 446 instances, the maintenance was not explicitly declared.

#### Interoperability: formality levels, languages and formats

The interoperability of semantic artefacts was evaluated by performing an analysis of formality levels, languages and formats (I1; mod:hasFormalityLevel; mod:hasRepresentationLanguage; mod:hasSyntax).

Semantic interoperability is empowered by clear definitions of terms and relations among them. More complex formality levels such as thesauri and ontologies with meaningful relations among terms, can help humans and systems to understand the context in which these terms are defined, and to retrieve the correct information. Overall, seven formality levels were found including ontologies (*N* = 199), thesauri (*N* = 153), glossaries (*N* = 145), gazetteers (*N* = 18), categorisation schemes (*N* = 13), subject headings (*N* = 7), and authority files (*N* = 5).

**Fig. 4:**
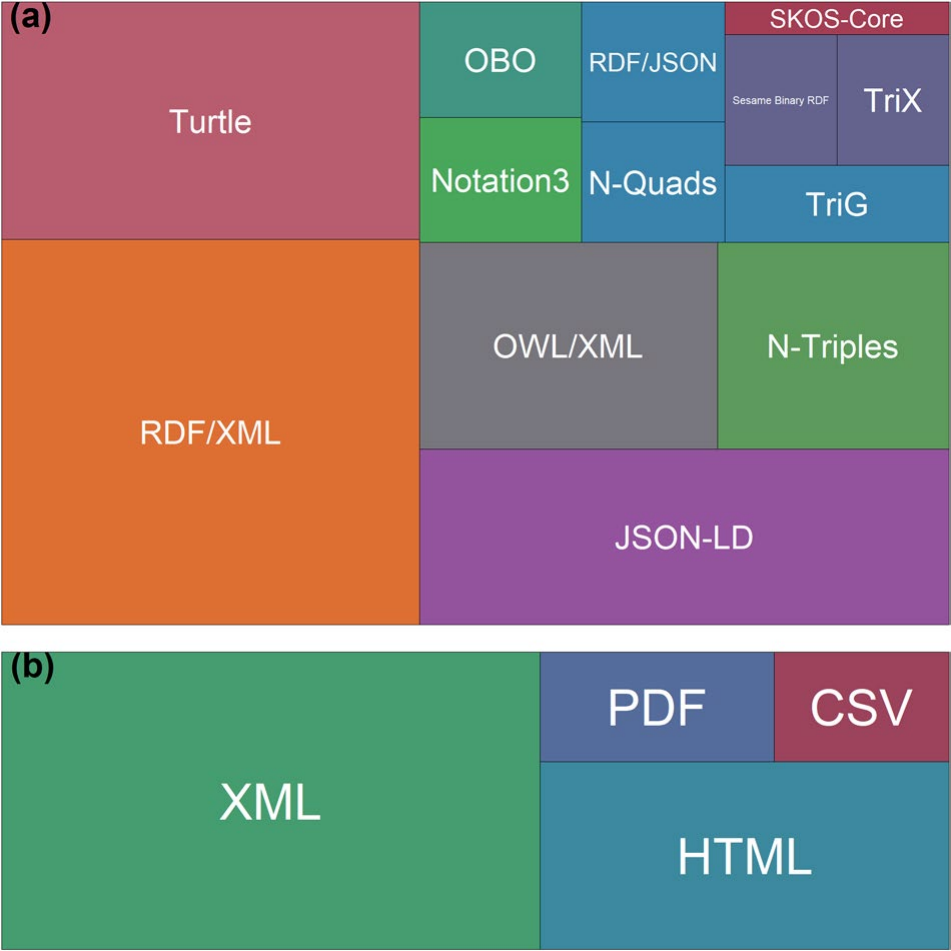
Treemaps showing the digital representation formats available for semantic artefacts built with standard models (**a**) and with no standard model (**b**). The box sizes are proportional to the number of formats.

These semantic artefacts were expressed in different languages and formats and, in most cases, they were available and downloadable in multiple formats (Fig. 4). A total of 497 semantic artefacts were built using standard languages and 43 were not. Specifically, 295 were built in SKOS, 201 in OWL, and one in RDFS^14^. The 497 semantic artefacts with a standard language were expressed as RDF/XML (*N* = 439; Fig. 4), Turtle (*N* = 271; Fig. 4), JSON-LD (*N* = 253; Fig. 4), OWL/XML (*N* = 168; Fig. 4), N-Triples (*N* = 131; Fig. 4), Notation3 (*N* = 55; Fig. 4), OBO (*N* = 51; Fig. 4), RDF/JSON, N-Quads, and TriG (*N* = 47; Fig. 4), Sesam Binary RDF and TriX (*N* = 40; Fig. 4), SKOS-Core (*N* = 20; Fig. 4) and other less common formats (*freq*  ≦  8). In addition, many of these artefacts were also available as CSV (*N* = 172) and PDF (*N* = 10). As for those semantic artefacts with no standard language (*N* = 43), 25 were available as XML, 12 as HTML, four as PDF, and three as CSV (Fig. 4).

#### Reusability: descriptions, usage licences and versions

The reusability was evaluated by analysing descriptions (R1; schema:description), usage licences (R.1.1; dct:licence) and versions available (R.1.2; pav:version).

Overall, 492 out of 540 semantic artefacts were published with a description or a brief abstract. Results of text mining analysis performed on descriptions showed that the most frequent terms were data (*freq* = 139), parameter (*freq* = 45), environment (*freq* = 44), plant (*freq* = 42), geoscience (*freq* = 37), trait and unit (*freq* = 36), development (*freq* = 35), anatomy, biological and marine (*freq* = 34), species (*freq* = 32), and biodiversity (*freq* = 31).

**Fig. 5:**
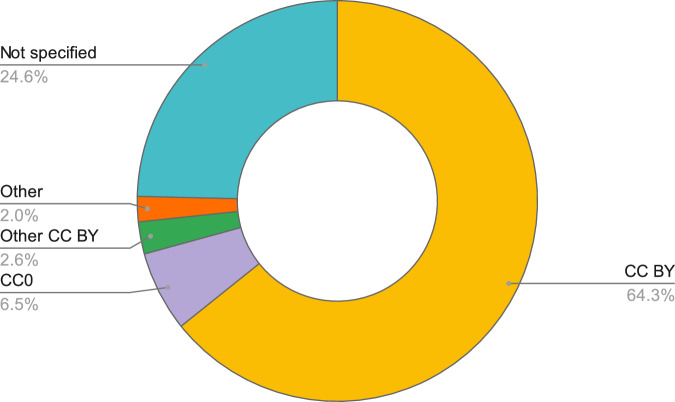
Donut chart with percentages of the different licence types of the semantic artefacts gathered within this study.

Usage licences were available for 407 semantic artefacts and, the majority of them (*N* = 347), were licensed under the Creative Common (CC) BY licence (64.3%; Fig. 5) and 35 were CC0 (6.5%; Fig. 5). In addition, 14 semantic artefacts were licensed under other types of CC BY licences (2.6%; Fig. 5) including CC BY-SA (*N* = 5), CC BY-NC-ND (*N* = 3), CC BY-NC-SA (*N* = 3), CC BY-ND (*N* = 2), and CC BY-NC (*N* = 1). Other types of licences were also found (2.0%; Fig. 5) including MIT (*N* = 3), BSD-3-Clause (*N* = 3), ODC-By (*N* = 2), and public licences (*N* = 2). Licences were not specified in 133 instances (24.6%; Fig. 5).

Version information was available for 419 semantic artefacts and different formats were used to present this information. The semantic versioning (*i.e*. MAJOR version.MINOR version.PATCH version^15^) was used in 273 instances, whereas dates were provided in 139 cases and, in seven cases, mixed formats (dates and semantic versioning) or alphanumeric strings (*i.e*. 3ed) were used.

### Management and governance

To evaluate the management of semantic artefacts accessible from multiple catalogues, a versioning analysis was carried out on those resources available in at least two of the 16 catalogues considered for this analysis (the I-ADOPT catalogue does not provide version information or the download option; Table 2). The version information was extracted for each semantic artefact and catalogue in which the resource was available. This collection was followed by the calculation of the versions’ alignment. In short, the number of times the resource had a unique value was counted and, such value, was then divided by the number of catalogues in which the resource and its version were available. When a semantic artefact had the same format and version across different catalogues, the resource was considered aligned. Conversely, if the version differed or it was expressed in a different format, the resource was considered misaligned.

**Table 2:** List of semantic catalogues and their exposed metadata. Black squares indicate the available metadata, white squares the metadata not available. Note: the “Title”, “Acronym”, and “Description” properties were not included in the table as they were available in all catalogues with I-ADOPT not showing the “Acronym” and “Description” properties. Refer to methods section for the full names of each metadata property.

Catalogue	URI	Identifier	Status	Deprecated	In catalogue	Formality	Language	Syntax	Licence	Version
AberOWL	□	□	□	□	□	□	■	□	□	■
AgroPortal	■	■	□	□	■	□	■	■	■	■
BARTOC	■	□	□	□	□	■	□	■	□	■
BioPortal	□	□	■	□	□	□	□	■	■	■
Bioregistry	■	□	□	□	■	□	□	□	■	■
NERC	■	□	□	□	□	□	□	■	■	■
EcoPortal	■	■	■	□	□	■	□	■	■	■
FAIRsharing	□	■	■	■	■	□	□	□	■	□
GFBio	■	□	□	□	□	■	■	□	■	■
I-ADOPT	■	□	□	□	□	■	■	□	□	□
LOTERRE	■	■	□	□	□	□	■	□	■	■
LOV	■	□	□	□	□	□	□	■	□	■
MMI-ORR	□	□	■	□	□	■	□	■	■	■
Ontobee	■	□	□	□	□	□	□	■	■	■
OBO Foundry	■	□	■	■	■	□	□	□	■	□
OLS	■	□	□	□	□	□	□	□	■	■
ARDC	□	□	□	■	□	□	□	□	■	■

**Fig. 6:**
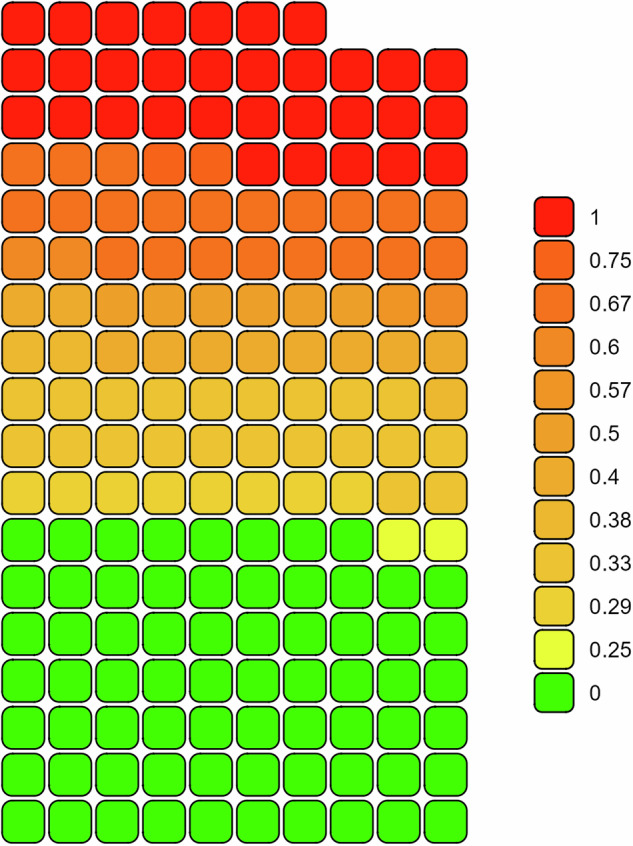
Waffle chart showing the versions’ misalignment of semantic artefacts available with version information on multiple (at least two) semantic catalogues. The misalignment is expressed as the proportion between the number of times in which each resource had a unique value and the number of catalogues in which the resource was available with a specified version. In the figure, each square represents a single semantic artefact (*N* = 177) and the proportion of misalignment varies from 0 (no existing misalignment among catalogues) to 1 (maximum misalignment among catalogues).

The versioning analysis was performed on 177 semantic artefacts (Fig. 6). In fact, of the 540 semantic artefacts collected, 207 resources were shared over at least two semantic catalogues and, only 177 of them, were available with version information and, hence, version comparisons could be performed.

Overall, 68 semantic artefacts had aligned versions across different catalogues (value = 0; Fig. 6) and, such resources, were shared between a minimum of two and a maximum of eight catalogues. A total of 109 semantic artefacts (61.8%; Fig. 6) had misaligned versions across the different catalogues in which such resources were available. Of the 109 misaligned semantic artefacts, 32 had the maximum misalignment (value = 1; Fig. 6) and were shared between two and three catalogues. The remaining resources had a misalignment proportion ranging between 0.25 and 0.75 (Fig. 6) and were available in between three and nine catalogues.

## Discussion

This study has brought forth a plethora of semantic artefacts within the environmental domain^14^. Despite their abundance, it is widely acknowledged that their adoption remains deficient or suboptimal, particularly within certain scientific communities^16^.

The limited use of semantic artefacts could be attributed to the lack of appreciation and understanding of their impact but also, to some extent, to their partial or absent compliance with established good practices and principles (*i.e*. FAIR principles), ultimately hampering the (re)usability of these resources^17^. The poor metadata descriptions (*e.g*. missing licences, versions, descriptions) and the lack of standards (*e.g*. languages and formats) coupled with inconsistencies of information provided by different semantic catalogues, exacerbates this challenge determining a failure to meet the semantic interoperability.

In this context, the main findings of this study are discussed to highlight the key issues limiting the FAIRness and the flawless management of semantic artefacts in the environmental domain, and to provide a glimpse of existing gaps and challenges that prevent the advancements toward the envisioned web of linked data^18^.

### Semantic artefacts in environmental sciences

This study performed an extensive analysis of 540 semantic artefacts in the environmental domain (Fig. 1, 3). These semantic artefacts have emerged independently, resulting in distinct models tailored and optimised for specific operations and scopes^2^. This evolution has led to the creation of semantic silos, potentially holding redundancies and overlaps.

The classification of semantic artefacts in environmental domains and the text mining analysis on titles and descriptions led to comparable results in terms of coverage and representation of environmental entities. These results showed that the majority of semantic artefacts described biological entities including anatomies, developmental stages, phenotypes, traits and taxonomies (Fig. 2) of specific taxonomic groups or species, many of which represented model organisms largely used in the experimental research. Many other semantic artefacts described cross-domain entities including parameters, environments, observations and methods (Fig. 2). These cross-domain semantic artefacts can be considered valuable resources for multidisciplinary projects and initiatives involving heterogeneous research communities, such as the ITINERIS project.

The frequency analysis resulting from text mining showed that only a limited number of terms had the highest frequencies. This result could indicate existing overlaps and topics overrepresentation and a more in-depth analysis would be required to support such findings. Furthermore, an investigation of the degree of alignments among terms could be used to measure the interoperability among these semantic artefacts. The overrepresentation in the biosphere domain might also have been generated by the adoption of the ITINERIS classification. According to ITINERIS, all organisms on Earth, independently of their habitats and ecosystems, are considered part of the biosphere. Nevertheless, the consistency of coverage analysis performed on domains and topics demonstrated that these resources represent a large portion of semantic artefacts in environmental sciences.

### FAIRness of semantic artefacts in environmental sciences

FAIR principles do not provide specific indications or implementation solutions as they are rather a set of guidelines to produce machine-actionable DOs^17^. While this flexibility has contributed to the rapid and widespread adoption of FAIR principles across different stakeholder communities, on the other side, it has generated a number of specific community-tailored solutions with the inherent risk of being incompatible among them^19^.

In this study, technical, technological and management choices related to the publication of semantic artefacts were analysed by examining core properties used to evaluate their FAIRness. The selected properties have been originally listed to design the framework of the O’FAIRe tool^20^, an online, metadata-based automatic FAIR assessment of semantic artefacts, already integrated into a number of semantic repositories^21,22^. Although the O’FAIRe tool allows a quantitative FAIR assessment of semantic artefacts, this can only be integrated in catalogues adopting the Metadata Ontology Description and publication (MOD)^23^ as metadata schema. The O’FAIRe score is computed based on the presence/absence of statements provided by the semantic artefacts’ creators rather than on the assessment of metadata content. The challenge of automated FAIR assessments lies in the inability of such services to generate unified evaluations based on the interpretation of different standards, formats and types of descriptors used within the metadata^24^. To overcome such limitations, this study focused on the evaluation of content and publication choices of semantic artefacts available across different or multiple semantic catalogues. Specifically, the analysis was performed on 13 core properties considered pivotal for the publication of FAIR semantic artefacts (Table 2). The analysis presented herein facilitated a broader and detailed evaluation of semantic artefacts distributed over multiple catalogues, each characterised by distinct metadata schemas and web interface visualisations^25,26^.

The analysis of persistent identifiers and the inclusion in semantic catalogues was used to evaluate the findability of semantic artefacts.

Thus far, multiple guidelines have been published on the construction of Persistent Identifiers (PIDs)^27,28^. By following the first FAIR principle (F1), both data and metadata of any DO should be identified with PIDs^29^. For semantic artefacts, this entails the assignment of PIDs to semantic artefacts and concepts/classes included therein as well as to their metadata^30^. This study focused on the collection of PIDs used to identify semantic artefacts (mod:URI) and DOIs minted to their metadata (dct:identifier). The results showed that only 18.5% of semantic artefacts metadata were assigned with DOIs, however such result depends on the management practices of semantic artefacts' catalogues in which the resources are available. For instance, semantic artefacts available in FAIRsharing are automatically assigned with DOIs^31^, and EcoPortal provides DOIs upon request^22^, whereas those available in OBO Foundry are identified with PURLs^32^. DOIs and PURLs are persistent identifiers, however, while PURLs can identify semantic artefacts, concepts/classes and their metadata, hence enabling machine interoperability, DOIs can only be minted to the resource landing page. Landing pages represent dead-ends for machines because it is not always clear which of the potentially many links available in the web page corresponds to the objects identified by the DOI^29,30^.

Semantic catalogues that do not have agreement with external services providing PIDs, use different types of URIs nevertheless ensuring the long term findability and accessibility of semantic artefacts and their metadata through their infrastructures. This study found that 5.5% of semantic artefacts were not included in any catalogue (Fig. 3) and, in a few cases, semantic artefacts were published in generalist data catalogues that do not offer the services to manage them (Fig. 3). In such cases, semantic artefacts are accessible through URLs of projects/initiatives-specific web pages. These resources face inherent challenges in terms of discoverability and sustainability, both in the short and long term^7^. This vulnerability is exacerbated by the risk of becoming lost when the hosting website/web page becomes no longer accessible or maintained.

Status and maintenance are considered key properties to assess the accessibility of semantic artefacts, however, this information was not provided for the majority of them. In addition, only a few catalogues formally report this information using different terminologies (Table 2). For example, some catalogues use software development status (*e.g.* AgroPortal, EcoPortal and BioPortal), whereas other catalogues adopt custom terminologies to describe the level of development of semantic artefacts (*e.g.* FAIRsharing, OBO Foundry), thus generating comparability issues and confusion for end-users. Overall, only 2,4% of semantic artefacts were no longer maintained, although this value could vary in time as it is strictly related to the responsiveness of semantic artefacts’ creators as well as to the promptness of catalogues’ updates^32^. Very often semantic artefacts are created *ad-hoc* for specific projects and, the lifespan of these resources is likely to be tight to the project duration. In this context, the role of semantic catalogues is fundamental to ensure the long term accessibility to these resources, even if they are no longer maintained, and to enable a persistent reference of those DOs that were annotated with them^8^.

The interoperability principle was analysed by considering formality levels, representation languages and formats. Predominantly, semantic artefacts comprised ontologies and thesauri, while the remaining resources consisted of sets of terms characterised by simpler structures and functions, such as glossaries, gazetteers, and categorisation schemes (Fig. 3). The observed variety of formality levels used to represent environmental concepts mirrors the heterogeneity of themes, levels of complexity, and scopes (Figs. 1, 2) for which these semantic artefacts were developed^33^. Ontologies were the most numerous component of this collection, primarily due to their capacity to precisely represent complex natural phenomena and entities such as environments, organisms and their interrelations^34^. Moreover, their ability to generate novel knowledge offers a significant advantage in the multidisciplinary field of environmental sciences as it facilitates and/or automatises new linkages and relationships in the networks of entities^33,35^. The majority of semantic artefacts were expressed as machine-actionable constructs, however, about the 8% of them lacked standard representation languages and formats (Fig. 4) and did not adhere to semantic web standards^36^. These semantic artefacts could be actively used by large communities that have invested significant time and effort in establishing agreed-upon terms. Transforming these resources into machine-actionable semantic artefacts holds the potential to amplify their reusability, concurrently limiting the duplication of efforts and minimising semantic overlaps if meaningful linkages are built^37,38^.

The reusability of semantic artefacts was examined by evaluating available descriptions, usage licences and versions. Descriptions, summaries or abstracts of semantic artefacts can help end-users in understanding the general scope, content and communities of reference for which the resource was conceived and developed^24^. Descriptions should be exhaustive in providing detailed information that can enhance their reusability, however, 8.9% of semantic artefacts were published without a description. The results of the text mining analysis performed on this property did not show significant differences with the one performed on titles, thus indicating that descriptions did not provide additional details. A further essential prerequisite to enable the reuse of existing semantic artefacts is the availability of clear usage licences^39,40^. Despite “usage licence” being listed among the minimum mandatory metadata for FAIR semantic artefacts^41^, 24.6% of the collected resources did not specify licences in their metadata (Fig. 5). The version is also a key aspect of semantic artefacts’ reusability whereby the semantic versioning is the recommended format to provide version information about semantic artefacts^42^. Semantic versioning enables the meaningful communication of the significance of a version change in the release of any DO^15^. The adoption of semantic versioning for the publication of semantic artefacts can facilitate, and in some instances, automate operations such as the diffing (*i.e*. the computation of changes between versions) and the retrieval of the most recent version by machines^42,43^. The study findings showed that 22.4% of semantic artefacts were published without version information while semantic versioning was used in 50.5% of cases and dates or other formats were used in the remaining instances. Another study, which focused on the analysis of ontologies indexed in OLS, also revealed a lack of version information or differences in version formats^24^. The study reported that only 2.5% of ontologies used the semantic versioning whereas the majority of them used date formats and 31.7% did not provide machine-readable versions^24^.

### Management and governance: current challenges and future perspectives

The storage and publication of semantic artefacts through semantic catalogues is highly recommended, however, those available in multiple catalogues can also generate management and governance challenges. An illustrative example is the existence of divergent versions of the same semantic artefact across different catalogues^10^. The versioning analysis found that 61.8% of the semantic artefacts available in at least two catalogues had distinct versions of the same resource (Fig. 6). This misalignment not only impacts the versions’ consistency but also affects other metadata properties evolving with it (*i.e*. identifiers, status, download links, relations to earlier versions, date and so forth)^42^.

Other inconsistencies, although not analysed here, can exist among catalogues. Such inconsistencies can be associated with different metadata properties and can generate confusion in end-user experiences as well as management issues when creating services for information exchange among systems. For example, when data collection occurred, discrepancies between catalogues were found in titles (*e.g*. the “Ontology of units of Measure” in OLS is the “Units of Measurement Ontology” in BioPortal; the “Core terms of Audubon Core” in LOV is the “Audiovisual Core Term List” in the GBIF Repository of Schemas), acronyms (*e.g*. the “Plant Trait Ontology” is PTO in BioPortal and TO in FAIRsharing and in OLS; “PATO” is the “Phenotypic Quality Ontology” in BioPortal and the “Phenotype And Trait Ontology” in OBO Foundry) and status (the “Anatomical Entity Ontology” is “Inactive” in OBO Foundry, in “Production” in BioPortal and “Ready” in FAIRsharing).

The community of practitioners, including developers and maintainers of semantic artefacts’ catalogues, are currently working on possible solutions to mitigate these challenges, such as in the case of the OntoPortal Alliance^10^. The consortium involves several research institutions and infrastructure teams dedicated to the development of semantic repositories in various disciplines based on the open and collaboratively developed OntoPortal software^10^. Within this consortium, developers and managers of the nine existing catalogues constantly strive to advance FAIR solutions for both semantic artefacts and their catalogues. This commitment involves the adoption of shared management and governance practices^44^, including efforts to minimise the replications of semantic artefacts across different repositories or to ensure the alignment of resources in instances where replications exist^10^.

The FAIR-IMPACT project is a further notable example of such efforts and it includes different tasks and work packages focusing on metadata, ontologies and semantic interoperability^45^. The project members are actively working on mapping existing FAIR assessment methods and developing a unique and integrated methodology for a consistent quantitative evaluation of semantic artefacts^46^. This methodology will enable a pre-publication assessment of semantic artefacts and the comparison of FAIR assessments performed with different tools, overcoming some of the challenges that have emerged within this study. Furthermore, to overcome existing semantic silos, FAIR-IMPACT members are designing solutions for FAIR mappings. These mappings will be enriched with provenance information and other associated metadata allowing the seamless transfer of information through the assignment of specific URIs^47^.

### Recommendations and concluding remarks

This study presents a thorough analysis of semantic artefacts within the environmental domain available online and through different catalogues. This analysis cannot be considered comprehensive due to the continuous release of novel semantic artefacts and catalogues. A noteworthy instance is the official launch of BiodivPortal and EarthPortal, featuring environmental semantic artefacts that hold relevance for both this study and the ITINERIS project. Such repositories were launched after the conclusion of the collection and analysis, and as such, they were not incorporated into the current study. However, the list of semantic artefacts^14^ should not be seen as a static document, but rather as a dynamic product capable of ongoing updates and integration in response to the publication of novel and pertinent resources.

This study, the collection of semantic artefacts, and all its new versions that may be published in the future, serve as a point of reference within the ongoing ITINERIS project, as well as for related initiatives and the broader environmental research community. In response to the gaps identified here, ITINERIS has established a task group on semantic interoperability and allocated funding to provide technical support and solutions to foster semantic interoperability among environmental RIs and beyond. With this study, the task group has laid the groundwork for the upcoming project developments which will consist in the publication of novel FAIR semantic artefacts, and in the extension and/or FAIRification of the existing ones. In addition, a terminology service is going to be designed and developed by taking advantage of the collection and assessment of semantic artefacts and catalogues performed in this study. The ITINERIS terminology service is conceived as a mediator between users, either humans or machines, and providers of semantic artefacts. Such mediation will facilitate the search and discovery of FAIR semantic artefacts and their meaningful links. These developments will foster the reuse of semantic artefacts, hence, avoiding overlaps and replications, and will improve their interoperability by bridging alignments among them.

To preemptively address semantic inconsistencies, adopting this approach is not just advisable but imperative, as already widely acknowledged at national and international levels^26^. This avenue represents the most meaningful way to enable FAIR science in practice and to achieve the envisioned full machine interoperation in the semantic web.

## Methods

**Fig. 7:**
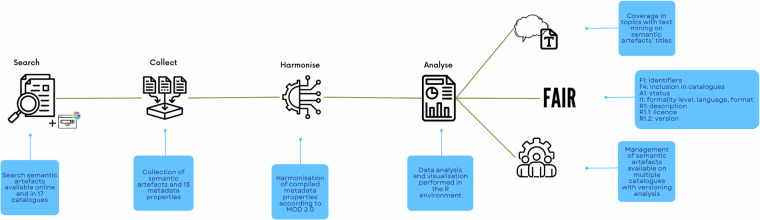
Collection and analysis of semantic artefacts. The diagram shows the collection of semantic artefacts and their metadata using 17 catalogues and a Google search. The “MOD 2.0” was used to harmonise the metadata properties analysed in the study. The analyses included an evaluation of topics’ coverage, FAIRness and management.

The collection of semantic artefacts (Fig. 7) was mainly performed through a search within 17 semantic repositories and registries listed in Table 1. These catalogues were selected as they offer access to a wide array of semantic artefacts relevant to different environmental domains including the atmosphere, marine, terrestrial biosphere and geosphere land surface domains of the ITINERIS project. The catalogues with less than 500 semantic artefacts were thoroughly examined either manually through their web interfaces or by using APIs or SPARQL interfaces. The catalogues with a larger number of semantic artefacts (> 500) were browsed by performing manual queries and filtering by specific keywords or relevant subjects/domains (check Table 1 for the number of semantic artefacts included in each catalogue). In addition to this preliminary investigation, the Google search engine was used to retrieve further semantic artefacts not included in the catalogues listed in Table 1. The Google search was performed using keywords such as “semantic resource” or “semantic artefact” together with environmental domain-specific keywords such as “marine” or “atmosphere” or “biosphere” or “geosphere” or “land surface”. During the collection, the semantic artefacts representing subsets of a main resource were not included in the list (*e.g*. the SWEET Ontology subsets *Material Element*, *Property Space Direction*, *Material Rock* contain classes belonging to the main SWEET ontology), whereas extensions were added (*e.g*. the SAREF4ENVI is a declared extension of the SAREF Ontology and includes different classes). The selected semantic artefacts were finally listed in a spreadsheet^14^. The collection of semantic artefacts included the harmonisation of the compiled metadata properties (Fig. 7). In fact, the catalogues adopt different metadata schemas and different labels to describe properties of semantic artefacts in their web interfaces. Considering such heterogeneity, the compiled properties were harmonised using, whenever possible, the labels from the Metadata for Ontology Description and publication (MOD v.2.0), and the following properties were collected:

- Title: A name given to the resource.
- Acronym: Often used as an identifier within some ontology platforms such as BioPortal or OBO Foundry.
- Description: A description of the item.
- URI: The URI of the ontology which is described by this metadata. When available, resource identifiers (*i.e*. URI or PURL) were used. If no resource identifier was available, the web page URL was added.
- Identifier: An unambiguous reference to the resource within a given context. This property was filled with the DOIs of the semantic artefacts when available.
- Status: The tracking information for the contents of the ontology.
- Deprecated: Specifies if the semantic artefact IRI is deprecated.
- Included in data catalogue: A data catalogue which contains this dataset. This property includes a list of all catalogues considered within this study (Table 1) and in which the semantic artefacts can be found.
- Has formality level: The level of formality of the semantic artefact (*e.g*. ontology, thesaurus, etc.). If not specified in the exposed metadata (Table 2), the formality level was assigned according to the classification described in previous studies^9,35,48^. For those semantic artefacts stating the formality level either in the title or in the description, the data models and formats were checked and, in case of inconsistencies, the formality level was adjusted according to the chosen classifications^9,35,48^. In addition, the semantic artefacts classified in the original description under the general umbrella term of “vocabulary” were checked to identify the most suitable formality level following the above-mentioned classifications.
- Has representation language: A language that is used to create an ontology.
- Has syntax: The syntax followed in the creation of the semantic artefact (*e.g*. XML, XML Schema, JSON, RDF/XML, OWL/XML, JSON-LD, Turtle, N-Triples, etc.). For each semantic artefact, all formats available across the different catalogues and web pages were extracted.
- Theme: A main category of the resource. The ITINERIS classification of the four environmental domains (*i.e*. atmosphere, marine, terrestrial biosphere, geosphere land surface) was used to categorise the shortlisted resources.
- Licence: A legal document giving official permission to do something with the resource. When not provided in the exposed metadata, licences were retrieved from the downloaded file of each resource. In case the semantic artefact was not provided in any downloadable format, the web page was searched and, if the licence could not be found, “Not Specified” was used as a value.
- Version: The version number of the resource. The most recent version of each semantic artefact was included. In addition, a further search was performed to gather and compare the resource versions across all catalogues in which the same resource was available to assess eventual discrepancies.
- Source accessed on: The resource is related to a source which was originally accessed or consulted on the given date as part of creating or authoring the resource.

Table 2 provides an overview of the exposed properties considered within this analysis for the 17 semantic catalogues used to compile the list of semantic artefacts.

Data analysis and data visualisation were performed in the R-Studio interface, R engine version 4.3.0^49^ (Fig. 7).

Data manipulation was carried out with the packages *dplyr* v.1.1.2^50^, *tidyverse* v.2.0.0^51^, *tidyr* v.1.2.0^52^, and *reshape* v.21^53^. To find the most frequent topics covered by the semantic artefacts gathered herein, a text mining analysis was carried out on the full title of the semantic artefacts and their descriptions by using *tm* package v.0.7-11^54^. The package *wordcloud* v.2.6^55^ was used to display results of text mining analysis performed on titles. To clean the text, the functions *removePunctuation*, *removeNumbers*, *stripWhitespace*, and *content_transformer* within *tm* were used, respectively, to eliminate punctuation marks, numbers, and extra white spaces and to convert all letters to lowercase. The *tm* function *removeWords* was applied to remove stopwords and common words such as semantic, ontology, thesaurus, vocabulary, glossary, terms, and terminology as well as project-specific words such as NERC, Argo, SeaDataNet etc.

As for the versioning analysis, only the semantic artefacts shared with a specified version among at least two catalogues were considered. To calculate the versions’ alignment across semantic catalogues, the number of times in which the resource had a unique version value was counted and, such value, was then divided by the number of catalogues in which the resource was available. The resulting values ranged between 0 and 1, whereby 0 represented a perfect alignment between versions of the same semantic artefact across different semantic catalogues and 1 represented the maximum misalignment of versions, *i.e*. all catalogues in which the same resource was available had different versions of that semantic artefact. Notably, when version formats of the same semantic artefact were not directly comparable, versions were also considered misaligned (*e.g*. instances in which version number and date were used in different catalogues as versions of the same resource).

Lastly, images were plotted using *ggplot2* v.3.4.2^56^ and the packages *treemapify* v.2.5^57^. and *ggvenn* v.0.1.10^58^ were used respectively for the treemaps and the Venn diagram. The waffle chart was generated using *waffle* v.1.0.1^59^ and *hrbrthemes* v.0.8.0^60^.

## Data Availability

The list of semantic artefacts and associated metadata is available on OSF^14^ at: 10.17605/osf.io/axy3s.

## Code availability

The R code used for data analysis and visualisation is available within the project “Collection of semantic artefacts in the environmental domains” of the OSF repository^14^ at: 10.17605/osf.io/axy3s.
